# Challenges in managing a vagal schwannomas: Lesson learnt

**DOI:** 10.1016/j.ijscr.2018.10.025

**Published:** 2018-10-19

**Authors:** Norhafiza Mat Lazim

**Affiliations:** Department of Otorhinolaryngology-Head & Neck Surgery, School of Medical Sciences, Universiti Sains Malaysia, Health Campus, Kubang Kerian, Kelantan, 16150, Malaysia

**Keywords:** Vagal nerve, Schwannomas, Paraganglioma, Head and neck tumor, Surgical excision

## Abstract

**Background:**

Paraganglioma of head and neck is a rare tumor and vagal schwannoma is even rarer. The majority of patients with vagal schwannoma presents with a lateral neck mass. Its management is delicate as the need to confirm the diagnosis by histopathology via a biopsy is contraindicated. Here, is a case of a young female with vagal schwannoma complicated with hoarseness after the biopsy of the mass, which persists after extirpation of the tumor.

**Case description:**

A 22-year old lady presented with a history of a right neck mass for a 5-months duration. Clinical examination revealed a mass at level II neck region which measures 3.0 cm × 2.0 cm and it was mobile, non-pulsatile and had smooth surfaced. CT scan and angiogram showed that the mass arose between the carotid artery and vagal nerve and it was a highly vascular lesion. A CT scan-guided biopsy performed but complicated with neck hematoma and patient developed hoarseness. On follow up, her hoarseness persists and her tissue biopsy came back as schwannoma. She was counseled regarding surgery versus radiation for her treatment and she agreeable for surgery. Hence, surgical excision was performed and intraoperatively the mass visualized arising from the vagal nerve. Postoperatively however, her voice did not improve.

**Conclusion:**

Vagal schwannoma is a rare paraganglioma of head and neck and the best treatment is still controversial. Unnecessary investigation and procedure should be avoided in order to reduce morbidity as well as improves patient’s quality of life.

## Highlights

• Vagal schwannoma is a rare tumor of head and neck region and mostly present with a slow growing mass at lateral neck.
• Even though it is a benign tumor, there is a predilection for malignant transformation, thus surgical extirpation is necessary in selected cases.
• Final diagnosis is obtained by tissue biopsy and in most cases, it is supplemented by details of clinical presentation and investigation finding.
• Biopsy of the mass can be complicated with hematoma and hoarseness, hence reduce patient’s quality of life.

## 1 Introduction

Paraganglioma of head and neck is a rare tumor and accounting for only 1% of all head and neck tumor. There are several types of paraganglioma that occurs mostly in the neck which includes glomus tumor, carotid body tumor and schwannoma. The majority of patient however is asymptomatic as the tumor is benign and has indolent growth. Clinical presentation depends on the types and locations of the tumor and the surrounding neurovascular structural involvement. Temporal bone paragangliomas or glomus tympanicum tumor normally originates in the middle ear, either from the Jacobson’s or Arnold’s nerves and characteristically shows bluish mass behind the tympanic membrane. The patient with glomus tympanicum tumor may present with a complaint of hearing loss, tinnitus and disequilibrium [1]. On the other hand, carotid body tumor grows within bifurcation of the carotid artery and generally present with a painless, mobile and slow growing mass over the neck region. This tumor type is commonly pulsatile and transmits the bruit.

Schwannomas of the neck is a rare type of paraganglioma and patient may present with neck swelling. If the schwannomas arise directly from the vagal nerve, the patient may be complaining of hoarseness or sometimes cough on palpation of the neck mass. In addition, with sympathetic nerves schwannoma, the clinical examination will reveal Horner’s syndrome which is characterized by ptosis, mydriasis and pupillary constriction or meiosis on the affected side. The final diagnosis of schwannoma is confirmed by a tissue biopsy, but it is not usually favored as it can give rise to complications of bleeding, hematoma and hoarseness.

The standard management approach for paraganglioma includes surgery, radiation, or watchful observation especially if it is asymptomatic. The symptomatic lesions may be justified for treatment of surgical resection if the expertise is available and consented by the patient. Of note, current trends have shifted to a more conservative approach for managing paragangliomas in view of its benign behavior with indolent growth and causing minimal symptoms. Head and neck paragangliomas may also occur as a sporadic or hereditary tumor. If it occurs as a hereditary trait, the paraganglioma may occur in association with sympathetic catecholamines-secreting paragangliomas, located in the abdomen or in the chest and they may occur as multiple tumors [2]. This report has been conducted in line with SCARE criteria [3].

## 2 Case history

**Fig. 1:**
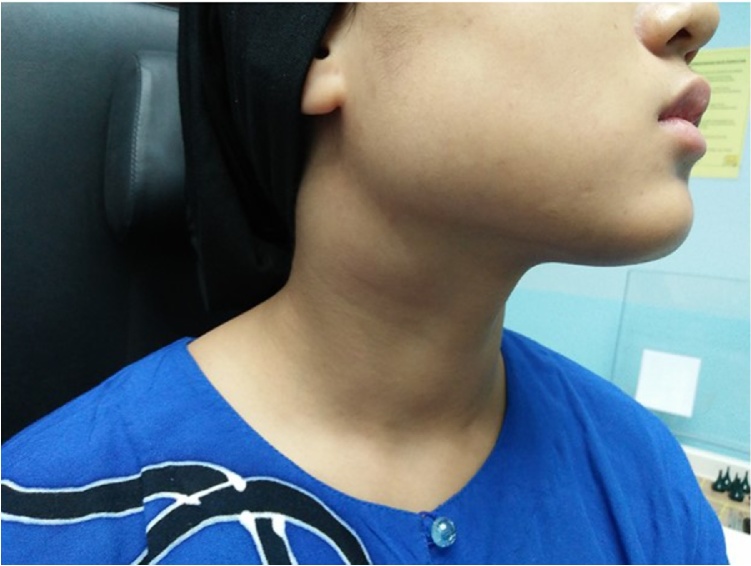
Clinical examination revealed a mass at right level II of the neck which measures 3.0 cm × 2.0 cm, rounded, mobile, non-pulsatile with smooth surfaced.

**Fig. 2:**
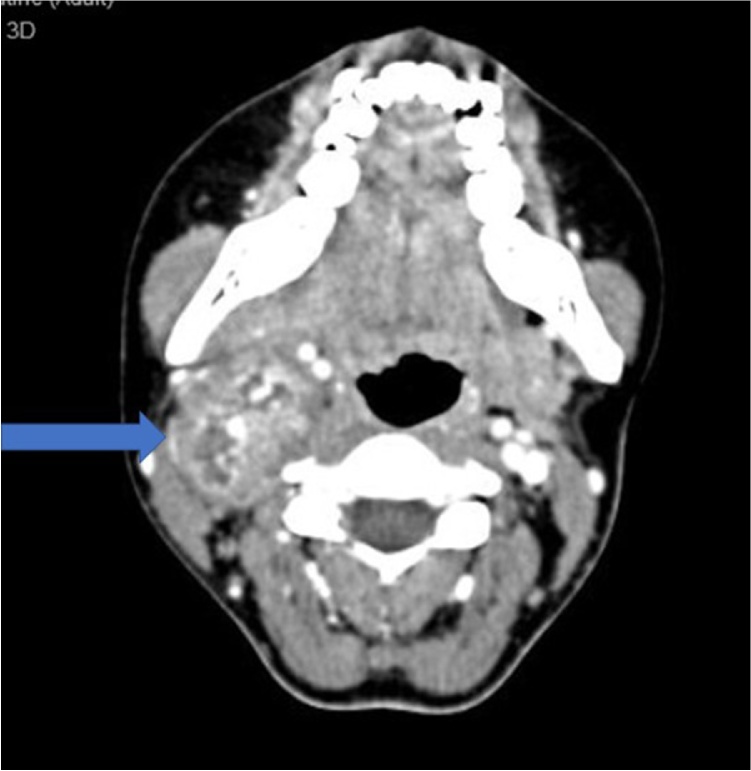
CT scan of the neck showed a heterogenous, well encapsulated tumor which located between the carotid artery and the vagal nerve.

A 22-year old Malay girl presented in early 2013 with a small right cervical neck swelling. Investigation performed at that time revealed a mass measuring 3.0 cm × 4.0 cm and it was a highly vascularized tumor on the angiogram. The patient was offered for treatment with radiation or surgery, however, patient refused and defaulted treatment. In 2015, she represented with increasing neck swelling associated with mild pain. There was no other significant complaint. Clinical examination revealed a mass at level II neck node measuring 3.0 cm × 2.0 cm which was mobile, non-pulsatile and had a smooth surface (Fig. 1). After discussion with an interventional radiologist, the patient was decided for a tissue biopsy under CT scan-guided and possibly with embolization in view of its vascularity. Subsequently, the patient underwent the biopsy under CT scan-guided but post procedure patient develops hematoma at the biopsy site and hoarseness. This hematoma had caused compression on the vagal nerve and caused recurrent laryngeal nerve paralysis with resultant hoarseness. A few days later, the patient was improved and was discharged home well and on subsequent follow up, it was noted that the neck hematoma has subsided but hoarseness persists. Endolaryngeal examination revealed a right vocal cord palsy with a minimal phonatory gap. The tissue biopsy results came back as schwannomas. Repeated CT scan showed the tumor was located between the carotid artery and the vagal nerve and it is a well-defined tumor with good demarcation from the surrounding tissue (Fig. 2). After lengthy discussing with the patient and her family, the patient was agreeable for surgery.

**Fig. 3:**
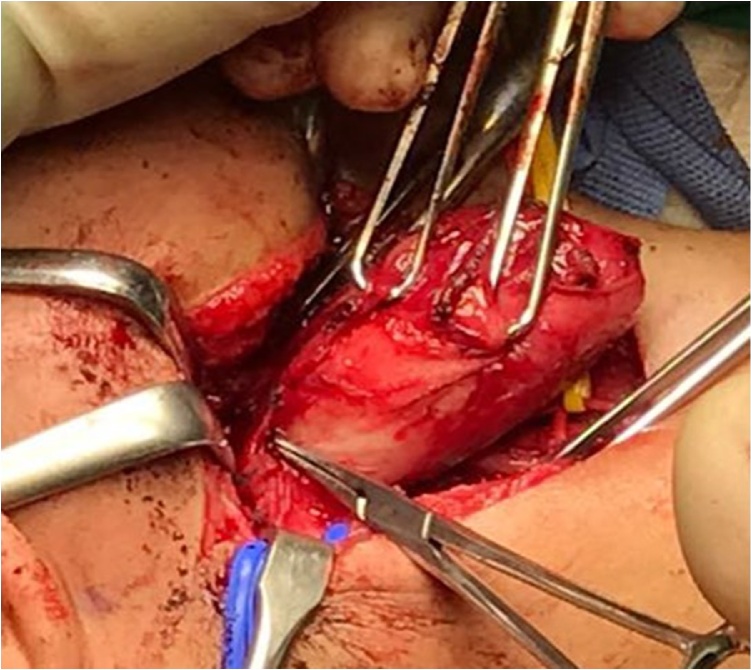
Intraoperatively, the mass was observed arising directly from the vagal nerve, in between the carotid artery and internal jugular vein.

**Fig. 4:**
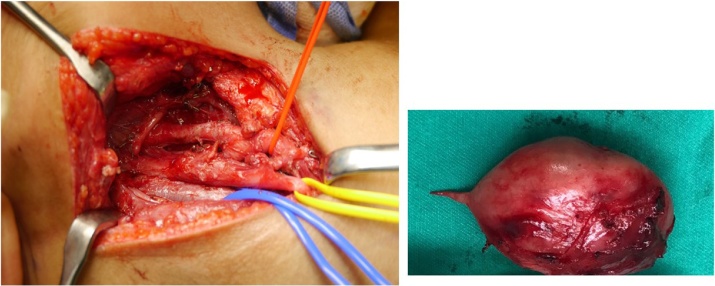
The mass was removed in total and measures 5.0 cm × 4.0 cm, leaving the vagal nerve intact (identified with yellow vessel loupe) with carotid artery and internal jugular vein (identified with red and blue vessel loupe respectively).

Intraoperatively, the patient was placed in supine position with slight neck extension and head turn to the contralateral side. The lesion and surgical anatomy landmarks were drawn on the patient skin, taking account preservation of the marginal mandibular nerve and expected cosmesis as well as ease of access to the carotid sheath. The skin incision performed and the skin flap was raised superiorly and inferiorly. The anterior border of the sternocleidomastoid muscle is skeletonized and exposing the mass deep to the sternomastoid. Continues dissection was performed and later, the carotid artery was identified and retracted laterally. The internal jugular vein was visualized deep and medial to the mass and was slowly retracted medially. The vagal nerve is difficult to identify initially but after deeper and careful dissection, the mass was observed arising directly from the perineurium of the vagal nerve (Fig. 3), in between the carotid artery and internal jugular vein. With gentle and meticulous dissection, the vagal nerve is separated from the mass. The mass was removed in total and measured 5.0 cm × 4.0 cm (Fig. 4), leaving the vagal nerve intact. The homeostasis was secured, and the wound closed in two layers with a small drain secured in-situ. The patient was discharged home uneventfully except for her hoarseness. The post-operative tissue biopsy result came back as vagal schwannomas and patient was doing well on the last follow up. Repeated laryngoscopy showed her right vocal cord remain paresis with partial compensation and minimal phonatory gap.

## 3 Discussion

Paraganglioma is a benign tumor and in the head and neck region and it constitutes about 1% of all tumors. In most cases, it is a slow-growing tumor and rarely causes severe symptoms. Details clinical presentation, however, depends on multiple factors such as the size of the tumor and its location. The standard management for head and neck paraganglioma includes close observation, surgical excision, radiation or stereotactic radiosurgery and in selected cases, it requires a combination of treatment. Both surgery and radiation may provide a good control of local tumors but with different complications which depend on the tumor extent and location. It has been documented that this paraganglioma may be associated with a malignant transformation especially in selected cases of mass with a long-standing history. On the other hand, Gilbo et al. divided the paraganglioma into benign and malignant type [1]. The clinical suspicion of malignant transformation may also be derived from its clinical features, development of metastases and complaint of pain.

The presentation of paragangliomas can be subtle. Interestingly, numerous clinical presentation and symptoms have been reported in literature ranging from hoarseness, sensorineural hearing loss, Horner’s syndrome, neck mass, and hypoglossal nerve palsy. The commonest symptom, however, is depending on the location and size of the tumor. In this case, the patient had a neck mass that progressively increases in size and hoarseness which developed after tissue biopsy strongly suggested that the mass was in close proximity to the vagal nerve. If surgical excision was decided for her treatment, the morbidity of the surgery would be less, as hoarseness already presents prior to the surgery. The aim of the surgery, however, is to preserve the integrity of the vagal nerve and its function. Imperatively, in this case, her hoarseness improving gradually postoperatively with no other complaints like history of aspiration.

According to Stambuk et al., most lesions arising in the parapharyngeal space are benign and therefore are well-defined. Small lesions arising from the parapharyngeal space are recognizable by the presence of fat around their periphery rather than displacement of the fat. Presence of an ill-defined lesion of the parapharyngeal space should raise suspicion for malignancy. The imaging features additionally will distinguish a paraganglioma from a carotid body tumor. He also documented that heterogeneity within a lesion is more commonly seen in schwannomas because of cystic change and hemorrhage. The schwannomas commonly present as a well-encapsulated tumor with a round or ovoid mass that is isointense to muscle on T1-weighted images, hyperintense on T2-weighted images, and enhances following contrast administration [4]. Importantly, the carotid body tumor also occupies the parapharyngeal space, and it tends to splay the internal and external carotid arteries. The paragangliomas often have demonstrable feeder vessels that most commonly arising from the ascending pharyngeal artery.

According to Smith et al., vagal paragangliomas were the biggest tumor in their study cohort with the size of 5.3 cm (±1.9 cm). They further mentioned that the most common mode of treatment is surgical excision with preoperative embolization. The resultant complications include complete unilateral vagal nerve paralysis together with additional glossopharyngeal, hypoglossal nerve, and cervical sympathetic chain disturbances [5]. Heyes et al. suggested that surgical excision is the standard treatment of choice for most vagal schwannomas but they stressed that nowadays, the contemporary management evolves toward a conservative modality due to high morbidity of the surgery. They further stated that vagal schwannomas resection almost always requires vagal nerve scarification with resultant speech, swallowing and sensory deficits [6].

Watchful waiting with serial imaging also has been successfully employed. Generally, most of the lesions remain radiologically stable in the majority of cases. In a few selected cases of schwannomas, there was some development and progression of neuropathy observed. Watchful waiting is especially valuable in asymptomatic older patients. This watchful waiting policy has its advantageous of preventing the surgery and its potential complications [7,8]. This is specifically true if the mass is small and does not cause any symptoms. The subsequent treatment of schwannomas also depends on the final tissue diagnosis. The surgical excision is advocates in any symptomatic cases with progressive disease as well as if the suspicion of malignancy is high in selected cases. The downside of watchful waiting is that even though the tumor is small, it can be found to be a malignant tumor and has a propensity for distant metastases, hence the best treatment is surgical excision. This crucial point explains why in this case we proceed with tissue biopsy even though we know it is a highly vascularized tumor, since the mass has been growing in size for more than three years. If the biopsy result came back as a malignant tumor, the treatment will be different where radiation should also be incorporated after surgical excision in her treatment plan. The adjuvant radiation is indicated if there are surgical margin involvement, perineural or perivascular invasion or high-grade malignancy.

Numerous surgical approaches for paraganglioma have been documented in the literature, and it mainly depends on the location of the tumor and its surrounding structural involvement. For the neck paraganglioma, the transcervical skin incision is opts with a suplatysma flap. This will adequately expose the tumor after meticulous dissection. The identification and preservation of the carotid artery and the internal jugular vein, as well as the vagal nerve is paramount. In this case, the carotid artery and internal jugular vein were retracted medially and laterally respectively to get a better surgical plane surrounding the tumor. Intraoperatively, in this case, the tumor arises from the perineurium of the vagal nerve as it is closely adherent to the vagal nerve. The vagal nerve together with hypoglossal nerve was preserved.

The other approach of the tumor can be transtemporal, transoral, transmandibular and combination of these approaches. The transmandibular approach is suitable for deep seated paraganglioma especially the one that located in the parapharyngeal spaces. In selected cases, the mandibulotomy has to be performed and the facial nerve needs to be identified, preserve and mobilized laterally. A transtemporal approach is used for glomus tympanicum and glomus jugulare tumors and can be combined with other approaches in order to completely remove the tumor. The surgical approach for glomus jugulare tumor can be combined with bicoronal craniotomy in order to excise the tumor in monobloc fashion [8,9]. The complications from surgery can be multiple, and it mainly depends on the experiences of the attending surgeons as well as the availability of multidisciplinary expertise in order to avoid unwanted surgical morbidities. These complications can be minimized in the expert hands and the outcomes of postoperative can be improved. This is imperative for the patient’s quality of life as in this case the patient is still young and there is a long path ahead so preserving the voice and aesthetic for her is paramount.

The other treatment modality for head and neck paraganglioma includes fractionated radiation and stereotactic radiosurgery. According to Smith et al., this treatment approach was most commonly employed for the jugular and vagal paragangliomas. In their study, they found out that the radiation produces excellent tumor control for 2–119 months with no major treatment complications [3]. These treatment types are best suited for large or multifocal head and neck paragangliomas who have a high likelihood of surgical morbidity to last four cranial nerves and also for those who are contraindicated for surgery due to medical comorbidities. Gilbo et al. stated that fractionated radiation dose required to treat benign paragangliomas is 45 Gy at 1.8 Gy per once daily fraction. The higher dose provides no improvement of local control and results in an increase in complications [1].

Most genetic studies on paraganglioma patients have highlighted the germline mutations in the RET, NFI and SDH mutations. According to Piccini et al., the gene mutations responsible for head and neck paraganglioma occurs mostly in genes encoding the subunits of succinate dehydrogenase or mitochondrial complex II which includes SDHD which located on 11q2 chromosome [3]. They revealed that a positive family history, the presence of multiple head and neck paragangliomas and association with sporadic paragangliomas were invariably characterized by a germ line mutation. The SDH mutations have been associated with clinical paraganglioma syndrome. Gilbo et al. documented that individual who is more likely to have SDH mutations are those who present with a family history of paragangliomas, a previous phaechromocytoma, multiple head and neck paragangliomas, age less than 40 years old and a male [1]. Genetic testing for hereditary head and neck paragangliomas is important as it will allow screening those family members and individuals at high risk as well as for mitigating life-long surveillance protocol.

## 4 Conclusion

Vagal schwannoma at the cervical region is an important clinical entity. The primary objective of any treatment strategy is to alleviate the patient’s symptoms and to optimize post-operative outcomes. Thus, unnecessary investigation and procedures such as biopsy should be avoided in order to maintain patient’s functioning and at the same time to achieve a better quality of life.

## Conflict of interest

I declare that I have no conflict of interest.

## Funding

There is no funding source involved in this case.

## Ethical approval

Not Applicable. This is only a case report not a research.

## Consent

Patient had given full consent for all data, material and photos for the purpose of publication.

## Author contribution

Only corresponding author is the primary and main author for this case report.

## Registration of research studies

Not applicable.

## Guarantor

Norhafiza Mat Lazim.

## Provenance and peer review

Not commissioned, externally peer reviewed.
